# Bio-ethical considerations in the application of artificial intelligence in spinal surgery

**DOI:** 10.1016/j.bas.2024.104161

**Published:** 2024-12-10

**Authors:** B. Bhavani Sowndharya, C.M. Mathan Muthu, A.S. Vickram, A. Saravanan

**Affiliations:** Saveetha Institute of Medical and Technical Sciences (SIMATS), Chennai, TN, India

Dear Editor,

Artificial intelligence is a developing tool in spine surgery, presenting both opportunities and bioethical challenges. This letter elucidates significant challenges regarding transparency, informed consent, data protection, accountability, and prejudice while debunking misconceptions about AI and highlighting its essential advantages without undermining ethical considerations. AI constitutes a collection of tools rather than an autonomous decision-maker. Current AI systems, such as a range of machine learning models, work under set parameters and do not make independent judgments. While their complexity may lead to “black-box” opacity, it does not inherently undermine patient autonomy. Patients must be educated on the role of AI as a supporting system in decision-making, so that true informed consent can occur, with decisions still resting with human clinicians (Quinn et al., 2022). It is vital to distinguish between Natural Intelligence, Artificial Intelligence (AI), and Artificial General Intelligence (AGI). AGI capable of human-like reasoning and decision-making remains speculative and requires solving profound concerns regarding consciousness and cognition. In contrast, contemporary AI systems are specialized tools designed to supplement, not replace, the decision-making processes of natural intelligence (Ray, 2024). This distinction underscores that patient autonomy and informed consent remain intact since AI systems are incapable of coercing patients or surgeons into making decisions. Clear communications about treatment alternatives and related risks are the foundation of informed consent. Awareness of the role of AI in analytical capacities is crucial when bringing AI into spinal surgery; nonetheless, the physician remains the final decision-maker (Solanki et al., 2023). AI-recommended answers should be presented as insights coming out of huge data analyses instead of a sure-shot solution. This alone assures that patients' freedom and their decision-making ability are maintained (Yanisky-Ravid et al., 2022). Handling patient data appropriately is a step toward continued trust. Observing standards such as the WHO's guidelines on digital health preserves sensitive data. Protecting the data to be used, stored, and shared combined with strict respect to the requirements for privacy will serve to lessen the danger. These must be created with privacy-preserving approaches to ensure the security of patient data (Fagherazzi et al., 2020). Accountability still offers a huge difficulty, mainly when things go wrong, and AI plays a role in the bad outcome. The supplier makes decisions, not the AI system. The surgeon should be diligent in making assessments about AI recommendations utilizing his or her knowledge to support the patient's safety. Developers and healthcare facilities have responsibility for training, validation, and maintenance of AI systems. A clear delineation of roles and responsibilities allows ethical and legal clarity. Bias in AI systems is expected to aggravate healthcare inequality. The training of AI on homogeneous datasets may lead to the creation of biased findings. Diverse quality datasets are thus necessary for the development of impartial AI technologies. Developers must also apply extensive validation techniques that discover and correct biases. In that manner, AI can assure fair and effective care for diverse sorts of groups of people who seek medical attention and eliminate inequities. While ethical considerations retain an important role, we cannot disregard the possibility of AI to improve medical practice. It provides insights into surgery planning and risk assessment in ways not achievable with enormous amounts of data (Upreti et al., 2024).

AI can present a holistic perspective of possible outcomes, thereby allowing clinicians to make useful decisions. For example, AI systems can detect complications, optimize surgical techniques, and increase patient outcomes by employing advanced analytics. Such benefits are evidence of AI as an addition to human skill, rather than a replacement (Spiegel et al., 2021). The ethical integration of AI in spine surgery would require substantial education and training for healthcare personnel. Surgeons need the ability to analyze AI outputs in-depth and include them properly in their practice. The capacity to make doctors adept in different domains-hence balancing surgical knowledge with AI knowledge drives the multidisciplinary care approach successfully (Daungsupawong et al., 2024). In spine surgery, AI presents difficulties and offers an opportunity. Though ethical issues concerning transparency, informed consent, data privacy, accountability, and prejudice are significant, these have to be evaluated against the potential AI holds to dramatically shift the locus of care. Addressing this tough issue involves multidisciplinary collaboration between innovators, providers of care, and policymakers. The main goal is to make AI supporting rather than isolating for the benefit of everyone involved while respecting their rights as patients and guaranteeing equality in providing care.

## Ethical approval

None.

## Funding source

Not Applicable.

## Declaration of competing interest

The authors declare that they have no known competing financial interests or personal relationships that could have appeared to influence the work reported in this paper.

## References

[bib1] Daungsupawong H., Wiwanitkit V. (2024). Harnessing artificial intelligence in bariatric surgery: correspondence. Surg. Obes. Relat. Dis..

[bib2] Fagherazzi G., Goetzinger C., Rashid M.A., Aguayo G.A., Huiart L. (2020). Digital health strategies to fight COVID-19 worldwide: challenges, recommendations, and a call for papers. J. Med. Internet Res..

[bib3] Quinn T.P., Jacobs S., Senadeera M., Le V., Coghlan S. (2022). The three ghosts of medical AI: can the black-box present deliver?. Artif. Intell. Med..

[bib4] Ray P.P. (2024). Artificial general intelligence for neurosurgery and medicine. J. Clin. Neurosci..

[bib5] Solanki P., Grundy J., Hussain W. (2023). Operationalising ethics in artificial intelligence for healthcare: a framework for AI developers. AI Ethics.

[bib6] Spiegel J.M., Ehrlich R., Yassi A., Riera F., Wilkinson J., Lockhart K. (2021). Using artificial intelligence for high-volume identification of silicosis and tuberculosis: a bio-ethics approach. Ann. Global Health.

[bib7] Upreti R., Lind P.G., Elmokashfi A., Yazidi A. (2024). Trustworthy machine learning in the context of security and privacy. Int. J. Inf. Secur..

[bib8] Yanisky-Ravid S., Fenster J. (2022). Decoding DaVinci: a novel approach to accountability and liability for medical devices operated through artificial intelligence based on" AI work made for hire" model. Intellect. Property J..

